# Symmetric private information retrieval supported by quantum-secure key-exchange network

**DOI:** 10.1038/s41377-022-00996-1

**Published:** 2022-10-14

**Authors:** Shuang Wang

**Affiliations:** 1 CAS Key Laboratory of Quantum Information, University of Science and Technology of China, 230026 Hefei, China; 2 CAS Center for Excellence in Quantum Information and Quantum Physics, University of Science and Technology of China, 230026 Hefei, China; 3 Hefei National Laboratory, University of Science and Technology of China, 230088 Hefei, China

**Keywords:** Quantum optics, Quantum optics

## Abstract

Quantum key distribution provides a provably secure way for private key distribution, which enables the practical implementation of information retrieval that preserves both the user privacy and database security.

Based on the fast-growing communication and networking techniques, retrieving information from a database is now a ubiquitous service that has a broad application prospect. For example, using a search engine to find out nearby restaurants, watching a movie on a streaming platform, etc. However, this task becomes non-trivial when there are privacy concerns, i.e., the user does not want to reveal his/her selection to either the data center or a third party, at the same time the data center does not want to reveal more information about the database other than the requested entry.

In 2000, Gertner and his colleagues proposed symmetric private information retrieval (SPIR) protocol that provides security guarantees to both the user and the database^1^. However, its practical implementation turns out to be experimentally demanding, requiring private random strings shared among parties. This is technically cumbersome with classical key distribution schemes based on computational complexity, and may not be suitable for applications requiring long-term security.

Quantum key distribution (QKD), whose security is based on the laws of quantum mechanics, is able to provide secret key distribution among distant parties with information-theoretic security. Since the first QKD protocol proposed by Bennet and Brassard in 1984, the field of QKD has developed extensively in both theory and experiment. With commercially available components and well-developed implementation techniques, QKD is now the most mature subfield of quantum cryptography^2,3^. Commercial QKD systems are also currently available on the market from several companies. Compared to its classical counterpart, the security of keys from QKD is independent of future advances in either hardware or algorithm, which is preferable for information retrieving tasks with data requiring long-term security.

Now, writing in this issue of *Light: Science & Applications*, Chao Wang and colleagues at the National University of Singapore report for the first time an experimental realization of SPIR supported by a measurement-device-independent (MDI) QKD network^4^. The SPIR demonstration looks at biometric security, and successfully retrieved a 582-byte fingerprint file from a database with 800 entries.

In the work presented here, the authors adopted a two-layered scheme for the system implementation, the SPIR application layer, and the MDI QKD layer (Fig. 1). The design of these two layers is based on the considerations of practicability and implementation security of the whole system. For the application layer, the authors adopted a two-database SPIR protocol^5^, since information-theoretically secure SPIR with a single data center is proven to be impossible^1^. For the QKD layer, they deployed MDI QKD^6,7^ with decoy states^8,9^. It has two main advantages. First, in MDI QKD, each party holds a quantum transmitter and communicates with each other via a central quantum receiver, which is operated by an untrusted third party. Thus, MDI QKD provides an appealing feature of immunity against any potential side-channel attacks on the quantum receiver, which is typically regarded as the most vulnerable part in practical QKD implementation. As such, each party only needs to secure their own transmitter and need not worry about the implementation of the quantum receiver. Second, MDI QKD holds a natural star topology, making it suitable for network expansion.

**Fig. 1:**
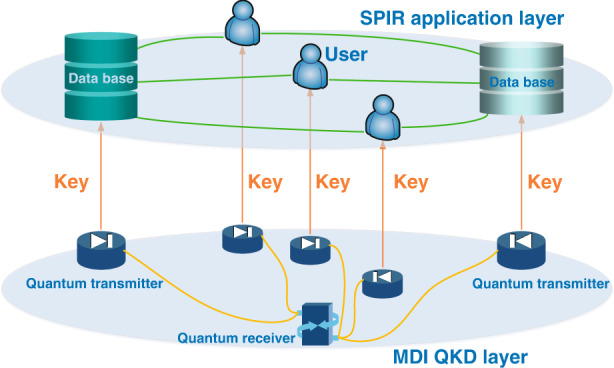
Schematic view of QKD-based SPIR. The symmetric private information retrieval (SPIR) application is supported by a measurement-device-independent quantum key distribution (MDI QKD) network

With the presented work, Chao Wang and co-authors set up a fiber-based MDI QKD system with a working frequency of 125 MHz. The quantum transmitter held by the user and data centers prepares time-bin phase encoding quantum states, where the information is coded on the intensities and the relative phase of the two successive temporal modes. Then, the quantum states are sent to the quantum receiver for measurement via an untrusted optical fiber channel with a length of 25 km. By carefully matching all the degree of freedoms of the incoming photonic quantum states, the authors obtained a Hong–Ou–Mandel interference visibility of 0.48 (±0.015), showing an efficient Bell-state measurement for the MDI QKD. They also measured the averaged bit error rate in the key generation basis to be 0.83%. After obtaining raw key bits, the authors performed classical post-processing, including error correction and privacy amplification, to obtain the final secure keys of 6.5 × 10^5^ bits. Finally, with the final keys, SPIR over a fingerprint database is successfully demonstrated.

The successful realization of SPIR supported by MDI QKD demonstrates the feasibility of the proposed scheme, providing a promising approach for the practical implementation of SPIR with information-theoretic security. With the recent advances of QKD in terms of high-speed^10,11^, long-distance^12–15^, and network optimization^16–18^, we can expect that the performance of the proposed scheme can be further promoted, pushing its practicability to a higher level. This will open the door to many applications where long-term security is critical, such as medical record retrieval, biometric authentication, and pay-per-use online contents, that would remain secure against even quantum computing based attacks.
